# Toward attention-based learning to predict the risk of brain degeneration with multimodal medical data

**DOI:** 10.3389/fnins.2022.1043626

**Published:** 2023-01-18

**Authors:** Xiaofei Sun, Weiwei Guo, Jing Shen

**Affiliations:** ^1^Department of Electrical and Electronic Engineering, The University of Hong Kong, Hong Kong, Hong Kong SAR, China; ^2^EchoX Technology Limited, Hong Kong, Hong Kong SAR, China; ^4^Department of Radiology, Affiliated Zhongshan Hospital of Dalian University, Dalian, Liaoning, China

**Keywords:** risk prediction of brain degeneration, multimodal medical data, multimodal learning, self-attention mechanism, cross-attention mechanism

## Abstract

**Introduction:**

Brain degeneration is commonly caused by some chronic diseases, such as Alzheimer’s disease (AD) and diabetes mellitus (DM). The risk prediction of brain degeneration aims to forecast the situation of disease progression of patients in the near future based on their historical health records. It is beneficial for patients to make an accurate clinical diagnosis and early prevention of disease. Current risk predictions of brain degeneration mainly rely on single-modality medical data, such as Electronic Health Records (EHR) or magnetic resonance imaging (MRI). However, only leveraging EHR or MRI data for the pertinent and accurate prediction is insufficient because of single-modality information (e.g., pixel or volume information of image data or clinical context information of non-image data).

**Methods:**

Several deep learning-based methods have used multimodal data to predict the risks of specified diseases. However, most of them simply integrate different modalities in an early, intermediate, or late fusion structure and do not care about the intra-modal and intermodal dependencies. A lack of these dependencies would lead to sub-optimal prediction performance. Thus, we propose an encoder-decoder framework for better risk prediction of brain degeneration by using MRI and EHR. An encoder module is one of the key components and mainly focuses on feature extraction of input data. Specifically, we introduce an encoder module, which integrates intra-modal and inter-modal dependencies with the spatial-temporal attention and cross-attention mechanism. The corresponding decoder module is another key component and mainly parses the features from the encoder. In the decoder module, a disease-oriented module is used to extract the most relevant disease representation features. We take advantage of a multi-head attention module followed by a fully connected layer to produce the predicted results.

**Results:**

As different types of AD and DM influence the nature and severity of brain degeneration, we evaluate the proposed method for three-class prediction of AD and three-class prediction of DM. Our results show that the proposed method with integrated MRI and EHR data achieves an accuracy of 0.859 and 0.899 for the risk prediction of AD and DM, respectively.

**Discussion:**

The prediction performance is significantly better than the benchmarks, including MRI-only, EHR-only, and state-of-the-art multimodal fusion methods.

## 1. Introduction

With the advent of artificial intelligence (AI), many deep learning–based methods (Schlemper et al., 2019; Zhang et al., 2019; Ye et al., 2021) using medical data have emerged as essential tools for aiding the early identification of disease severity. Commonly, medical data can be divided into two broad modalities: image data, such as magnetic resonance imaging (MRI) and computed tomography (CT), and non-image data, such as Electronic Health Records (EHR).

Brain degeneration is a chronic brain disease that disturbs the brain’s normal functioning and further brings a huge threat to public health (Pratico, 2008). Several research studies (Nicolls, 2004; Xu et al., 2009; Stanciu et al., 2020; Cheung et al., 2022) have revealed that adults with chronic diabetes mellitus (DM), including type 1 diabetes and type 2 diabetes, have a higher risk of developing AD. The severity and duration of DM could contribute to brain degeneration (Pruzin et al., 2018). Thus, AD becomes the most common cause of brain degeneration and typically begins with impairments in cognitive functions (Li and Hölscher, 2007). According to the different development of cognitive degradation, AD is divided into three stages, including the pre-clinical (e.g., cognitively normal) stage, mild cognitive impairment (MCI) stage, and dementia stage (Pratico, 2008). MCI is key to diagnosing the early stage of AD. Similarly, DM is classified as type 1 diabetes mellitus (T1DM) and type 2 diabetes mellitus (T2DM) depending on differences in diabetes mechanisms. Patients with T1DM and T2DM would present brain degeneration at different levels.

Many deep learning methods (Escott-Price et al., 2015; Moeskops et al., 2018; Li and Fan, 2019; Xu et al., 2020; Yang and Liu, 2020; Zhu et al., 2020) have been developed to predict the risk of brain degeneration from various aspects, e.g., the transition from MCI to AD in advance, and the cognitive impairment in patients with T1DM and T2DM. These risk prediction methods can effectively reduce the incidence rate of concurrent brain degeneration diseases. Because of a huge data domain gap between medical images and EHR, the difference in prediction accuracy is significant when using medical images or EHR, respectively. The medical images (e.g., MRI) present the vital anatomical information that non-image data (e.g., EHR) lack. EHR is regarded as an important auxiliary for accurate medical image interpretation, particularly for DM diagnosis (Biessels and Reijmer, 2014). Therefore, the fusion of medical images and EHR could provide sufficient information and improve prediction accuracy. Most deep learning–based methods (Li et al., 2019; Ljubic et al., 2020; Yang and Liu, 2020; Yiğit and Işik, 2020; Alexander et al., 2021; Zhang et al., 2021) for predicting the risk of brain degeneration from some chronic diseases only utilize single-modal data. The learnable features from single-modal data may suffer from serious biases of the learning model, which lack imaging or clinical context information. Several learning-based methods (Spasov et al., 2018; Li and Fan, 2019; Zhou et al., 2021) using medical images and EHR data have attempted to predict disease risk by a multimodal data fusion model. However, very few deep learning–based methods account for the inter-modal and intra-modal relationships and have been explored for better accurate risk prediction of brain degeneration.

Medical imaging datasets account for anatomical information and are insufficient to train a network alone. The main reason is the lack of clinical information that is embedded in the EHR dataset. It may lead to unbalanced classes and inaccurate prediction (Huang et al., 2020). EHR is a kind of hierarchical data that stores the historical health status of a patient in temporal sequences formed by multiple visits (Shickel et al., 2017). EHR data of a patient are usually represented by a sparse binary matrix. Only encoding a sparse vector in the deep learning–based method may cause a lack of diversity for potential embedding space, thus increasing the challenge for network training without large volumes of image data (Ye et al., 2020). Therefore, only leveraging EHR data for the risk prediction of brain degeneration is also insufficient.

To solve the above limitations, combining medical imaging with EHR data is necessary for compensating patients’ more detailed historical health status. More specifically, medical images, such as MRI, could offer more complex interpretations of a patient’s health status, thus leading to a more elaborate embedding space for potential risk-generation tasks. However, most deep learning–based methods (Shickel et al., 2017; Xu et al., 2020; Zhang et al., 2021) using multimodal data only integrate the medical data from different modalities in a simple manner, such as an early, intermediate, or late fusion structure. A lack of deep exploration of the intra-modal and inter-modal dependencies leads to sub-optimal prediction performance.

The attention mechanism (Vaswani et al., 2017) has emerged with the coming of transformer architecture. It is an input processing technique for neural networks that allows the network to focus on specific parts of a complex input, one at a time until the entire dataset is processed. Attention can provide the ability to highlight vital information and suppress irrelevant information. In the tasks of medical imaging analysis, the spatial–temporal self-attention mechanism (Schlemper et al., 2019; Chen and Shi, 2020; Chen et al., 2020; Plizzari et al., 2021; Yu et al., 2021; Mehta et al., 2022) is often used to capture the spatial and temporal correlations of the same image sequences. The cross-attention mechanism (Hou et al., 2019; Huang et al., 2019; Yu et al., 2021) can capture the interdependent relationship between two sequences of single-modal or multimodal data by integrating two separate embedding sequences with the same dimension asymmetrically. The attention has been effectively applied to medical image analysis to achieve promising results. Some deep learning–based studies (Wang et al., 2018; Jiang et al., 2021) only use simple concatenation for the combination of multimodal features after a feed of medical images (e.g., MRI, CT, or X-ray) and clinical context features (e.g., EHR). The attention mechanism can provide the ability to emphasis on important information and suppress irrelevant counterparts of multimodal features. However, the attention mechanism is scarcely adopted to capture the correlations between medical images and non-image data. The goal of this study is to solve the abovementioned problems. We thus develop a novel attention–based framework for predicting the risk of brain degeneration by making better use of medical images and EHR data. First, a spatial and temporal attention encoder is composed of a set of self-attention blocks. The encoder is employed to extract the complementary features information based on multimodal data to achieve the intra-modal dependencies. This encoder often helps extract the critical pixel information of MRI. Then, for gaining the inter-modal dependencies between MRI and EHR data, a cross-attention mechanism is used to extract the cross-correlation from these two modalities. After two attention encoders, we also propose to adopt the multi-head attention decoder for combining the features of different modalities before the final fully connected (FC) layer. The decoder can guarantee an optimal global feature representation depending on its powerful combination ability in different subspaces.

To sum up, the contribution of this study is two-fold. First, different from the previous multimodal fusion methods of varying medical data modalities (Arevalo et al., 2017; Huang et al., 2020; Jiang et al., 2021; Nagrani et al., 2021), we focus on extracting the critical complementary information between MRI and EHR data with the attention mechanisms for the prediction of brain degeneration. Second, multi-head attention as a disease-oriented decoder is used to improve the prediction performance to avoid sub-optimal issues. We perform the experiments on an available publicly Alzheimer’s Disease Neuroimaging Initiative (ADNI) dataset and an internally collected diabetes mellitus (DM) dataset to evaluate the performance of our proposed method.

## 2. Materials and methods

### 2.1. Materials

#### 2.1.1. Internally collected datasets

All internal data used in the study are collected from Zhongshan Hospital Affiliated with Dalian University. The protocol for this retrospective study was approved by the Ethics Committee of Zhongshan Hospital Affiliated with Dalian University. The requirement for written informed consent from study participants was waived.

The dataset includes 396 subjects with T1-weighted MRI and the corresponding EHR. A patient’s diagnosis in the internal data is classified as normal control (NC), T1DM, and T2DM. This study includes 99 NC cases, 135 T1DM cases, and 162 T2DM cases. The EHR data contain a total of 17 features [demographic information: age, gender, years in education; fasting glucose; glycated hemoglobin (HbA1c); triglyceride (TG); cholesterol (CHO); low-density lipoprotein (LDL); high-density lipoprotein (HDL); C Peptide; Montreal Cognitive Assessment (MoCA); clock drawing test (CDT); verbal fluency test (VFT); trial marking test A (TMT-A); anxiety level; depression level; and sleep quality]. The MRI data are directly used for all following experiments in this study to avoid information loss because of preprocessing operation. All 17 features of EHR data are considered to use in the following experiments.

#### 2.1.2. Public datasets

The data used in the evaluation of this study are obtained from Alzheimer’s Disease Neuroimaging Initiative (ADNI) database (Jack et al., 2008) for analyzing the progression of Alzheimer’s disease (AD). An essential goal of ADNI database is to evaluate whether medical images, including MRI and PET, and other modality EHR data including biological markers and clinical and neuropsychological assessment information, can be integrated to predict the AD progression from MCI or pre-clinical stage for accurate diagnosis and early prevention.

We select the training data according to the following rules (Jiang et al., 2021). For each patient, the first scanned MRI with description information “multiplanar reconstruction (MPR); GradWarp; B1 Correction; N3.” A patient’s diagnosis in the ADNI is typically classified as AD, MCI, and cognitively normal (CN). In this study, we select the whole data from 969 subjects, containing 288 AD cases, 365 MCI cases, and 316 CN cases. For each patient, one MRI sequence is accompanied by corresponding EHR data. The MRI data are also directly used in this study. The selected EHR data contain a total of 11 features [demographic information: age, gender, years in education, and ethnic and racial categories; biofluids: APOe4 genotyping; cerebrospinal fluid (CSF) levels; behavioral assessments: clinical dementia rating (CDRSB); Alzheimer’s disease assessment scale (ADAS13); the episodic memory evaluations in the Rey Auditory Verbal Learning Test (RAVLT_immediate); and The Mini-Mental State Examination (MMSE)]. All 11 features of EHR data are considered to use in the following experiments.

### 2.2. Methods

**FIGURE 1:**
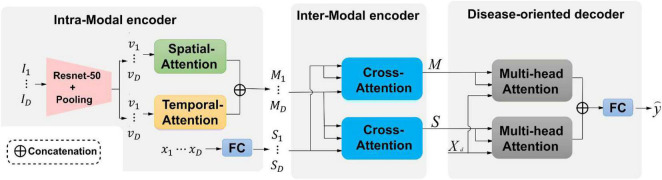
An illustration of the proposed framework for the risk prediction of brain degeneration. Our framework has two attention encoders and one decoder. {***I***_*i*_ ∈ ℝ^{*H* × *W*}^|*i* ∈ {1,…,*D*}} is an MRI sequence ***I***.{***v***_*i*_ ∈ ℝ^{*H* × *W*}^|***v*** ∈ {1,…,*D*}} is an MRI features representation. {***x***_*i*_ ∈ ℝ^{*H* × *W*}^|*x* ∈ {1,…,*D*}} is the corespongding Electronic Health Records (EHR) data of MRI. ***S*** and ***M*** are the feature representations across spatial-attention and temporal-attention, respectively. *X_d_* represents the classification query. $\hat{\text{y}}$ is the final prediction label according to the categories of diseases.

This study develops an end-to-end framework for predicting the risk of brain degeneration by taking in the complementary features between MRI and EHR data. The input data of the network are the paired MRI and EHR data. 3D ResNet-50 (Yu et al., 2021; Mehta et al., 2022) is the backbone network in the initial stage. Other deeper networks, such as DenseNet (Huang et al., 2017), also work with our proposed framework. The output is the prediction result, which is represented as binary values. To address the issues of the intra-modal and inter-modal dependencies, two attention mechanisms are deployed in the two-level encoder module. To be specific, self-attention as the first-level encoder, which includes spatial and temporal attention, is utilized to extract the spatial–temporal feature information for the internal-slice dependencies of the same MRI sequence. The EHR data and disease representations from the self-attention output are passed into the second-level cross-attention encoder. This encoder considers the inter-modal dependencies by extracting the correlations between the features from MRI and EHR data. After the encoder, the multi-head attention mechanism as a decoder aggregates the information from all dimensions for producing the final prediction. The overall network architecture of risk prediction of brain degeneration is shown in Figure 1.

Given the observed history of patient health status in multiple visits, an available visit is represented by {***I**_1_, **I**_2_, …, **I**_*D*_, **x***}, where {*I*_*i*_ ∈ ℝ^{*H* × *W*}^|*i* ∈ {1,…,*D*}} represents the *i*-th slice from an MRI sequence, *H* and *W* denote the height and width, respectively. Binary vector set ***x*** ∈ ^ℝ*D*^ is EHR data of each MRI sequence, each element in ***x*** belongs to {0,1}, where 1 denotes the presence of the corresponding AD and vice visa. The task needs to predict the risks of getting *K* categories of diseases, which could be represented as $\hat{\text{y}}\in {\left[0,1\right]}^{K}$. Our framework consists of two encoders that integrate intra-modal and inter-modal dependencies in a spatial–temporal manner and a disease-oriented decoder with multi-head attention to extract the most relevant disease representations.

#### 2.2.1. Intra-modal encoder

Given medical images, intra-modal dependencies are first generated by capturing the spatial–temporal relations of MRI modality in an independent module. Considering the MRI sequence {***I_1_**, **I_2_**, …, **I_D_***}, where *D* is the number of slices from one MRI sequence, a ResNet-50 and a spatial average pooling layer are adopted to extract the disease features representation {***v_1_**, **v_2_**, …, **v_D_***}, where each element is a *C*-dimensional vector with shape (1,*C*). After repeating the above operations for all MR slices of one visit, *C* × *D* vectors are separately processed by two blocks from spatial and temporal domains. As shown in Figure 1, one disease representation *v_i_*, which stands for the *i*-th slice, interacts with other representations in the spatial-attention block to capture the intra-slice relations. *v_i_* interacts with other representations in the temporal block to compute the inter-slice variations from the same MR sequence. Based on the MRI sequence, the relations between two continuous slices are retrieved with temporal attention, and the relations of pixels in one slice are retrieved with spatial attention. Both the two attention mechanisms follow the spatial and temporal structure as described in Mehta et al. (2022).

**FIGURE 2:**
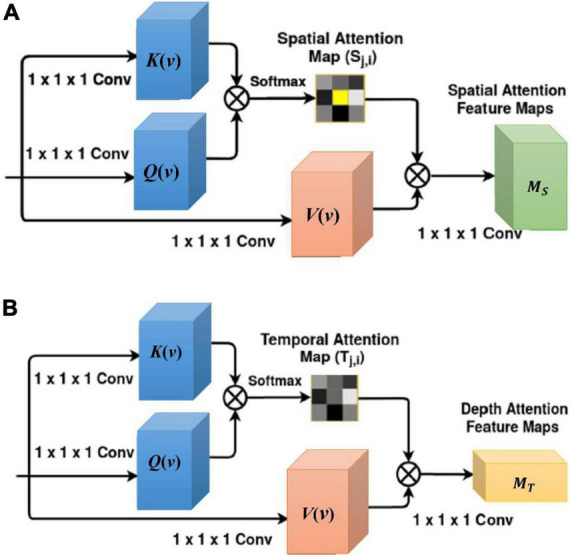
**(A)** 3D spatial-attention architecture; **(B)** 3D temporal-attention architecture. *K*(*v*) is the key, *Q*(*v*) is the query and *V*(*v*) is the value. ⊗ is defined as an element-wise multiplication operation. Noted that spatial or depth is an interchangeable term.

As shown in Figure 1, spatial attention is used to capture intra-slice dependencies. The relationships between each pixel and other pixels in the slice are computed. These relations are passed with dominant intra-frame dependencies. The illustration of spatial attention is shown in Figure 2A and mathematically expressed by the following equation:


(1) $$S_{j,i}=\frac{\exp\left(K\,{\left(v_{i}\right)}^{T}\,Q\,\left(v_{j}\right)\right)}{\sum_{i}^{H\times W}\exp\left(K\,{\left(v_{i}\right)}^{T}\,Q\,\left(v_{j}\right)\right)}.$$

The disease representation *v* through ResNet-50 and spatial average pooling layer is transformed to the key *K* (*v*_*i*_), query *Q* (*v*_*j*_), and value *V* (*v*_*i*_) by using 1 × 1 × 1 convolution filter. The relationships between pixels are represented by the spatial dimension (*H* × *W*) × (*H* × *W*). ***S**_**j**,**i**_* ∈ ℝ^{*C* × *D* × *H* × *W* × *H* × *W*}^ is spatial correlation matrix for computing the impact of *i*-th position on *j*-th position and obtained by softmax of the inner product of *K* (*v*_*i*_) and *Q* (*v*_*j*_). Here, *C* is the number of channels. The output attention features across spatial dimensions are written as:


(2) $${\hat{\text{M}}}_{S}=\sum_{i=1}^{H\times W}V\,\left(v_{i}\right)\,S_{j,i}.$$

Then, ${\hat{\text{M}}}_{S}\in {\mathbb{R}}^{\left\{C\times H\times W\times D\right\}}$ is fed into 1 × 1 × 1 convolution filter, which results in the final spatial-attention features ***M_S_*** with *C* channels.

The temporal attention captures an MRI sequence’s inter-slice dependencies and relates the global features between two slices of the same MRI sequence in the temporal domain. The illustration of temporal attention is shown in Figure 2B and mathematically expressed by the following equation:


(3) $${\text{T}}_{j,i}=\frac{\exp\left(K\,{\left(v_{i}\right)}^{T}\,Q\,\left(v_{j}\right)\right)}{\sum_{i}^{D}\exp\left(K\,{\left(v_{i}\right)}^{T}\,Q\,\left(v_{j}\right)\right)}.$$

The relationships between pixels are represented by the depth dimension *D* × *D*. ***T**_**j**,**i**_* ∈ ℝ^{*C* × *D* × *H* × *W* × *D* × *D*}^ is a dimensional temporal correlation matrix for computing the impact of *i*-th slice on *j*-th slice. The output attention features across temporal dimension are written as:


(4) $${\hat{\text{M}}}_{T}=\sum_{i=1}^{D}V\,\left(v_{i}\right)\,T_{j,i}.$$

Then, ${\hat{\text{M}}}_{T}\in {\mathbb{R}}^{\left\{C\times D\times H\times W\right\}}$ is fed into 1 × 1 × 1 convolution filter, which results in the final temporal-attention features ***M_T_*** with *C* channels.

For each spatial and temporal attention block, the final output is then concatenated along with the spatial dimension to form *D* matrices where each one owns the shape of (*D*, *C*). Finally, disease representations of medical images {***M***_*i*_ ∈ ℝ^*D* × *C*^|*i* ∈ {1,2,…,*D*}} are generated by summing matrices with the same visit index from different attention blocks. For the EHR vector sequence {***x***_1_, ***x***_2_, …, ***x***_*D*_} comprise of *D* time points for one MRI sequence, a fully connected layer is adopted to embed each EHR vector into a *C*-dimensional space to capture the overall health information by producing a vector with shape (1,*C*), which results in disease representations {***S***_1_, ***S***_2_, …, ***S***_*D*_} of EHR data.

#### 2.2.2. Inter-modal encoder

**FIGURE 3:**
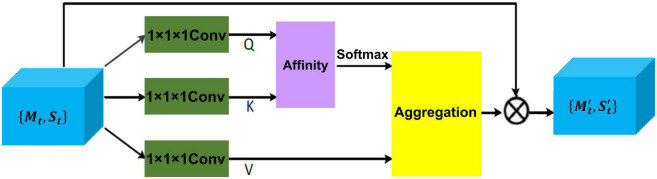
The details of cross-attention architecture to extract the cross-correlation of multimodal features. Q, K, and V represent all extracted feature maps. {***M***_*t*_, ***S***_*t*_} is the disease feature representation, and $\left\{{\text{M}}_{t}^{\prime },{\text{S}}_{t}^{\prime }\right\}$ is the final representation.

Inter-modal dependencies between MRI and EHR are captured through a cross-attention mechanism, which exchanges the global health status from EHR data and detailed disease information from MRI in a parallel manner. Given disease representation {*M*_*t*_, *S*_*t*_} for *t*-th slice, two cross-attention modules as shown in Figure 3 are leveraged to compute the cross-correlation of multimodal features by taking queries from their own modalities while key and value matrices from opposite modalities, which results in $\left\{M_{t}^{\prime },S_{t}^{\prime }\right\}$.

To be specific, disease representation *via* two 1 × 1 × 1 convolution filter produces two feature maps *Q* and *K*, respectively, where {*Q*,*K*} ∈ ℝ^{*C* × *H* × *W*}^. After obtaining *Q* and *K*, the feature attention maps are generated *via* affinity operation (Huang et al., 2019) and softmax.

At each position *j* in the spatial dimension of feature map *Q*, a vector *Q*_*j*_ ∈ ℝ^*C*^ is obtained. For the total features set Ω_*j*_ ∈ ℝ^(*H* × *W*−1) × *C*^ also can be obtained by capturing the spatial features vectors from feature map *K*, which are in the same row with position *j*. Here, Ω_*i*,*j*_ ∈ ℝ^*C*^ represents the *i*-th element of Ω_*j*_. The affinity operation is formulated as follows:


(5) Affi,j=Qj⁢Ωi,jT

where ***Aff**_**i**,**j**_* is the correlation degree between ***Q_j_*** and Ω*_**i**,**j**_*. Then, a softmax layer is applied on ***Aff**_**i**,**j**_* across each channel to calculate the attention map ***A*** from affinity operation.

Another 1 × 1× 1 convolution filter is applied to disease representation *H* ∈ {***M***_*t*_, ***S***_*t*_} to produce feature map *V*, the final representations {${\text{M}}_{t}^{{}^{\prime }},{\text{S}}_{t}^{{}^{\prime }}\}$ is obtained by aggregation operation (Huang et al., 2019) for achieving the mutual feature gains from MRI and EHR.

Similarly, at each position *j* in the spatial dimension of feature map *V*, a vector *V*_*j*_ ∈ ℝ^*C*^ and the total features set ${\hat{\Omega }}_{j}\in {\mathbb{R}}^{\left(H\times W-1\right)\times C}$ are obtained. Here, ${\hat{\Omega }}_{i,j}\in {\mathbb{R}}^{C}$ represents the *i*-th element of ${\hat{\Omega }}_{j}.$ The aggregation operation is formulated as follows:


(6) $$A\,g\,g_{j}=\sum_{i\in \left|{\hat{\Omega }}_{j}\right|}A_{i,j}\,{\hat{\Omega }}_{i,j}+H_{j}$$

where *Agg*_*j*_ is a feature vector at position *j*. *A*_*i,j*_ is scalar data, which belongs to affinity feature map *A*. The most relevant contextual information is added to local disease representation *H* to enhance the local features and augment the disease representation. Thus, these disease feature representations achieve mutual gains between MRI and EHR data.

After repeating the operations for each slice corresponding to an independent time point, *D* updated vectors of EHR are concatenated into ***S*** ∈ ℝ^*D* × *C*^, and a compressed disease representation of medical images ***M*** ∈ ℝ^*D* × *C*^ is produced by concatenating and pooling the $\left\{{\text{M}}_{1}^{{}^{\prime }},{\text{M}}_{2}^{{}^{\prime }},\ldots ,{\text{M}}_{D}^{{}^{\prime }}\right\}$ across the temporal dimension.

### 2.3. Disease-oriented decoder

Disease-oriented decoder seeks the most relevant information in two different modalities for predicting the risk of brain degeneration. The right part of Figure 1 shows that the decoder includes two multi-head attention layers and a fully connected layer. The multi-head attention layer expects disease representations ***M***, ***S***, and a classification query ***X_d_*** ∈ ℝ^*K* × *C*^ as input, where ***K*** is the number of disease risk categories included in the task. By conducting the multi-head attention mechanism, which follows the multi-head attention of the transformer (Vaswani et al., 2017), the most relevant clinical contextual information for brain degeneration is updated and stored in the query. Finally, the outputs of two multi-head attention layers are added together and transmitted into a fully connected layer to produce the final prediction result $\hat{\text{y}}\in {\mathbb{R}}^{K}$. Actually, the prediction risk of brain degeneration is a classification task, and the cross-entropy loss function is applied at the training stage to train the model.

## 3. Experiments and results

### 3.1. Implementation details

We implement our proposed method on Pytorch to classify three stages of AD progression, including CN, MCI, and AD. For the training stage, four Nvidia Tesla V100 GPUs with 32GB memory are used. We employ a polynomial learning rate policy where the initial learning rate is multiplied by $1-{\left(\frac{i\,t\,e\,r}{t\,o\,t\,a\,l_{i\,t\,e\,r}}\right)}^{p\,o\,w\,e\,r}$ with *power* = 0.9. The initial learning rate we used is set to 0.01. Momentum and weight decay coefficients are 0.9 and 0.0001, respectively. The input size of MRI is 256 × 256 × 170, the batch size is set to 32. Five-fold cross-validation is performed to split the training data. We perform 100 epochs of training for all settings. All the intensities of input MRI images are normalized to [0,1].

### 3.2. Results

#### 3.2.1. Evaluation metrics

Four evaluation metrics are calculated to evaluate the risk prediction performance on the test cases of internally collected DM datasets and ADNI datasets. These metrics include sensitivity, accuracy, specificity, and area under the receiver operating characteristic curve (AUROC). All the evaluation metrics are reported in the following ablation and comparison experiments.

#### 3.2.2. Ablation study for intra-modal and inter-modal encoders

We employ self-attention mechanisms, including a spatial-attention mechanism (SAM) and a temporal-attention mechanism (TAM) for the intra-modal encoder and a cross-attention mechanism (CAM) for the inter-modal encoder. The addition of these two encoders can contribute to capturing the intra-modal and inter-modal dependencies for better prediction. To verify the encoder module’s performance and analyze each component’s actual contribution, we conduct ablation experiments with different settings on both DM and ADNI datasets in Tables 1, 2.

**TABLE 1:** Quantitative results on internally collected diabetes mellitus (DM) datasets for the proposed method with or without the specified components.

Intra-modal encoder		Inter-modal encoder	Sensitivity	Accuracy	Specificity	AUROC
**SAM**	**TAM**	**CAM**				
			0.562 ± 0.016	0.596 ± 0.012	0.742 ± 0.012	0.714 ± 0.012
√			0.601 ± 0.012	0.634 ± 0.015	0.762 ± 0.011	0.772 ± 0.012
	√		0.719 ± 0.012	0.716 ± 0.013	0.766 ± 0.012	0.802 ± 0.012
√	√		0.762 ± 0.012	0.752 ± 0.016	0.771 ± 0.012	0.839 ± 0.011
		√	0.764 ± 0.012	0.784 ± 0.014	0.778 ± 0.012	0.842 ± 0.014
√		√	0.771 ± 0.016	0.801 ± 0.016	0.792 ± 0.011	0.861 ± 0.013
	√	√	0.834 ± 0.015	0.823 ± 0.015	0.816 ± 0.012	0.887 ± 0.012
√	√	√	**0.887 ± 0.016**	**0.859 ± 0.012**	**0.867 ± 0.012**	**0.916 ± 0.012**

The ‘√’ symbol represents the inclusion of components. The results from the proposed method with SAM, TAM, and CAM are highlighted in bold. SAM represents spatial-attention mechanism, TAM represents temporal-attention mechanism, and CAM represents cross-attention mechanism.

**TABLE 2:** Quantitative results on Alzheimer’s Disease Neuroimaging Initiative (ADNI) datasets for the proposed method with or without the specified components.

Intra-modal encoder		Inter-modal encoder	Sensitivity	Accuracy	Specificity	AUROC
**SAM**	**TAM**	**CAM**				
			0.536 ± 0.011	0.626 ± 0.013	0.755 ± 0.012	0.732 ± 0.012
√			0.588 ± 0.012	0.726 ± 0.015	0.772 ± 0.011	0.791 ± 0.013
	√		0.708 ± 0.013	0.826 ± 0.015	0.864 ± 0.013	0.897 ± 0.012
√	√		0.742 ± 0.012	0.833 ± 0.014	0.872 ± 0.012	0.909 ± 0.011
		√	0.802 ± 0.013	0.852 ± 0.015	0.878 ± 0.013	0.913 ± 0.013
√		√	0.841 ± 0.014	0.866 ± 0.016	0.893 ± 0.010	0.931 ± 0.012
	√	√	0.886 ± 0.015	0.885 ± 0.016	0.884 ± 0.013	0.936 ± 0.012
√	√	√	**0.901 ± 0.014**	**0.899 ± 0.013**	**0.892 ± 0.012**	**0.953 ± 0.013**

The ‘√’ symbol represents the inclusion of components. The results from the proposed method with spatial-attention mechanism (SAM), temporal-attention mechanism (TAM), and cross-attention mechanism (CAM) are highlighted in bold.

**FIGURE 4:**
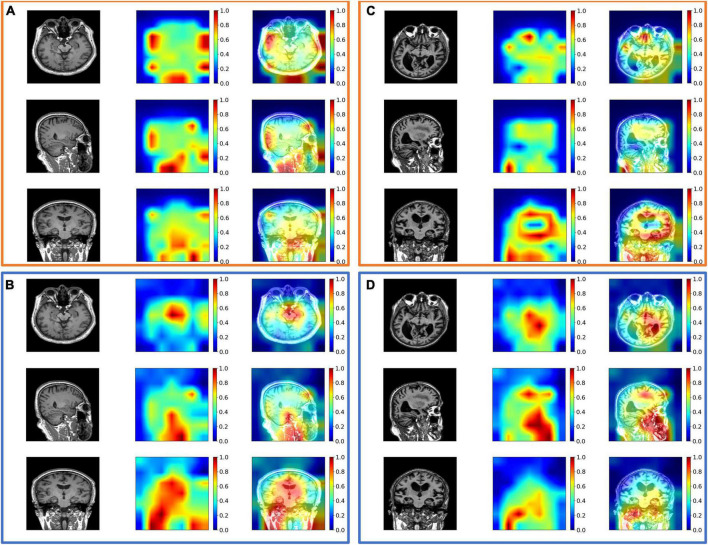
The exemplary attention maps **(A)** with spatial-attention mechanism (SAM) and temporal-attention mechanism (TAM) and **(B)** without SAM and TAM on diabetes mellitus (DM) datasets; **(C)** with SAM and TAM, and **(D)** without SAM and TAM on Alzheimer’s Disease Neuroimaging Initiative (ADNI) datasets. The views from the top row to the bottom row are axial, coronal, and sagittal views. The corresponding images from left to right are the original image, attention map, and image overlayed with the attention map. The value of the attention map from zero to one is assigned blue to red colors. Noted that attention maps without SAM and TAM may suffer from inaccurate feature extraction, such as high attention values close to 1 out of the head in panels **(A,C)**.

As shown in Tables 1, 2, the intra-modal and inter-modal encoders remarkably improve the prediction performance on internally collected DM and public ADNI datasets. The baseline method only uses the multi-head attention mechanism, as shown in the first row of Tables 1, 2. Compared with the baseline method, employing SAM and TAM in the intra-modal encoder achieved a significant prediction improvement with an accuracy of 0.762 on DM datasets and an accuracy of 0.742 on ADNI datasets. The visual attention maps in Figure 4 with SAM and TAM showed that the attention mechanism in the intra-modal encoder could capture the critical area (around the location of the hippocampus) features, which are quite relevant to brain degeneration. Only employing the CAM in the inter-modal encoder yields an accuracy of 0.784 on DM datasets and 0.852 on ADNI datasets, which are higher than the accuracies of only employing the SAM and TAM in the intra-modal encoder. Then, in our proposed method, we further combine the SAM and the TAM in the intra-modal encoder with the CAM in the inter-modal encoder, and the highest accuracies of 0.859 on DM datasets and 0.899 on ADNI datasets are achieved. In particular, on DM datasets, the proposed method outperforms the method with only an intra-modal encoder and the method with only an inter-modal encoder by 16.4 and 16.1%, respectively. We also observe that our proposed method achieves the best results for other evaluation metrics for both DM and ADNI datasets. Similarly, results substantiated that multimodal encoders considering intra-modal and inter-modal dependencies greatly benefit the risk prediction of brain degeneration based on different disease datasets (e.g., DM datasets and ADNI datasets).

#### 3.2.3. Evaluation of multi-head attention decoder

**FIGURE 5:**
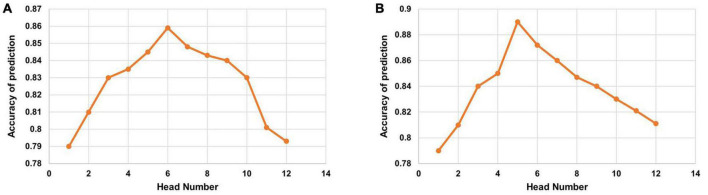
Accuracy of the multi-head attention with the varying head number on **(A)** diabetes mellitus (DM) datasets and **(B)** Alzheimer’s Disease Neuroimaging Initiative (ADNI) datasets.

After two encoders, we employ the two multi-head attention layers as a disease-oriented decoder. The multi-head attention mechanism with multiple head numbers can focus on the most relevant features from multimodal representation subspaces to reach an optimal global representation. We evaluate the multi-attention decoder in our method with varying head numbers for a comprehensive comparison. We evaluate the impact of the head number on the multi-head attention mechanism. As shown in Figure 5, the accuracy performance of multi-head attention with head numbers from 1 to 12 is evaluated on both DM and ADNI datasets. From the observation of Figure 5, when the head number reaches the optimal head number, the performance decreases with increasing head number values. It is observed that the head number is set to six for DM datasets, and the highest accuracy of risk prediction of brain degeneration is demonstrated. Similarly, as shown in Figure 5B, the head number is set to five for our used dataset from the ADNI database, and the highest accuracy is observed. It implies that the optimal head number may vary for different data domains due to the data domain gap (Liu et al., 2021).

#### 3.2.4. Comparison

We compare our method with MRI-only method-3D DenseNet (Ruiz et al., 2020), EHR-only method-ElasticNet (Zou and Hastie, 2005), and three typical learning-based multimodal fusion methods.

The MRI-only method only depends on the pixel information from MRI data for predicting the outcome. For our MRI-only method, we use the 3D DenseNet model (Ruiz et al., 2020), which utilizes MRI and is capable of considerable risk prediction of brain degeneration. The 3D DenseNet primarily consists of layers of 3D convolutions with skip connections.

The EHR-only method only depends on parsing the EHR data through preprocessing step. More precisely, the EHR data of a patient are usually denoted by a sparse binary matrix where each element is an International Classification Disease code (ICD-9) (Benesch et al., 1997) in a specified visit. Several learning-based methods (Ma et al., 2018; Zhang et al., 2019; Luo et al., 2020; Ahuja et al., 2021) have put effort into encoding the potential temporal relations, especially between distinct visits of EHR and output the risk prediction of disease through a multi-task paradigm. For our EHR-only method, we use an ElasticNet (Zou and Hastie, 2005) model, which takes in a concatenation of all EHR features.

In clinical practice, pertinent clinical information is vital for providing accurate diagnostic decisions during medical imaging interpretations (Boonn and Langlotz, 2009; Zhou et al., 2021). The fused feature maps from MRI and EHR data in our compared multimodal fusion methods are performed by (1) Early fusion (Spasov et al., 2018) based on concatenation; (2) intermediate fusion (Jiang et al., 2021) based on linear layers and (3) late fusion (Arevalo et al., 2017) based on single-head attention strategies.

The concatenation method is implemented by concatenating the pooled image feature and EHR feature at the input level. Different from the concatenation method, linear layers of a conventional neural network (CNN) mainly adopt a linear transformation for each modality data to obtain the transformed features with the same size for multimodal data. These two transformed features from medical image and EHR are added up to a fused feature. The fusion based on single-head attention is performed by employing standard attention as an aggregation strategy before the FC layer.

We use the ResNet-50 as the backbone for all methods and the same datasets to guarantee a fair comparison. We benchmark the performance of different methods on the entire test data using four different evaluation metrics. The results of the metrics are reported in Tables 3, 4 on DM and ADNI datasets. For both DM and ADNI datasets, we observe that the EHR-only method can achieve better performance than the MRI-only method for the risk prediction of brain degeneration on all the evaluation metrics. It means that EHR data could provide informative data for the clinical diagnosis of brain degeneration. When combining MRI and EHR data, the three multimodal fusion methods further enhance the prediction performance compared with the MRI-only and EHR-only methods. It proves that EHR is crucial for the complementary interpretation of MR images. Given the results of prediction performance from Tables 3, 4, late fusion works better for fusing MRI and EHR data to predict the risk of brain degeneration than early fusion and intermediate fusion. Unlike these three typical fusion methods, the proposed method considers the intra-modal and inter-modal dependencies for learning more modality-aware mutual and complementary features. These enhanced features lead to noticeable performance improvement on DM and ADNI datasets. Thus, the proposed method achieves the best results on all four evaluation metrics. Especially on ADNI datasets, the accuracy of 0.899 in our method is much higher than the accuracy of 0.757 in the worst MRI-only method, with a significant improvement of 18.7%.

**TABLE 3:** Performance comparison of the MRI-only method, the Electronic Health Records (EHR)-only method, the early fusion method, the intermediate fusion method, and the late fusion method on the test diabetes mellitus (DM) dataset.

Methods	Sensitivity	Accuracy	Specificity	AUROC
MRI-only	0.674 ± 0.012	0.763 ± 0.012	0.772 ± 0.013	0.742 ± 0.012
EHR-only	0.745 ± 0.012	0.818 ± 0.014	0.822 ± 0.012	0.832 ± 0.012
Early fusion	0.789 ± 0.013	0.825 ± 0.011	0.826 ± 0.012	0.841 ± 0.012
Intermediate fusion	0.827 ± 0.012	0.831 ± 0.014	0.839 ± 0.011	0.853 ± 0.012
Late fusion	0.841 ± 0.012	0.833 ± 0.012	0.851 ± 0.012	0.867 ± 0.011
**Proposed**	**0.887 ± 0.016**	**0.859 ± 0.012**	**0.867 ± 0.012**	**0.916 ± 0.012**

The bold values means the best performance among these methods.

**TABLE 4:** Performance comparison of the MRI-only method, the Electronic Health Records (EHR)-only method, the early fusion method, the intermediate fusion method, and the late fusion method on the test Alzheimer’s Disease Neuroimaging Initiative (ADNI) dataset.

Methods	Sensitivity	Accuracy	Specificity	AUROC
MRI-only	0.658 ± 0.013	0.757 ± 0.014	0.853 ± 0.011	0.843 ± 0.014
EHR-only	0.786 ± 0.012	0.829 ± 0.016	0.866 ± 0.013	0.903 ± 0.012
Early fusion	0.806 ± 0.014	0.852 ± 0.013	0.877 ± 0.012	0.913 ± 0.013
Intermediate fusion	0.815 ± 0.012	0.851 ± 0.015	0.875 ± 0.011	0.910 ± 0.012
Late fusion	0.873 ± 0.014	0.882 ± 0.014	0.886 ± 0.012	0.928 ± 0.011
**Proposed**	**0.901 ± 0.014**	**0.899 ± 0.013**	**0.892 ± 0.012**	**0.953 ± 0.013**

The bold values means the best performance among these methods.

## 4. Discussion

The main novelty of the proposed method is to incorporate the correlated features between MRI and EHR data into a global disease representation in a tightly coupled way, which depends on the attention mechanisms in intra-modal and inter-modal encoders. To further emphasize the impact of each attention component, the ablation experiments are performed by the single addition or the combined addition of different attention mechanisms to the baseline method. Our proposed method has the highest predictive ability to distinguish the three levels of brain degeneration progression, which occur in DM and AD patients, respectively. This is mainly because our method preserves the high correlation between MRI and EHR data by capturing intra-modal and inter-modal dependencies. Notably, our method adds spatial–temporal attention and cross-attention to capture the intra-modal dependencies of an MRI sequence. The intra-modal dependencies provide sufficient anatomical features and significantly improve the prediction. The visualization of the different attention maps is shown in Figure 4. For the DM dataset, we can observe the SAM and TAM that can emphasize the critical brain area, which implies the features of the critical area are more relevant to the classification of DM patients. As for the ADNI dataset, the SAM and TAM can also focus on the critical brain area, such as the area around the hippocampus.

In addition to finding a method that can capture the intra-modal and inter-modal dependencies, there is an important need to seek the most relevant features to avoid sub-optimal prediction performance. Following that, we employed two multi-head attention layers to project the inputs into multiple different subspaces to a more elaborate embedding space for the final prediction. Because of different head numbers, the effectiveness of multi-head attention may vary. To reach the optimal performance, Figure 5 shows that larger head numbers do not bring a consistent increase in the prediction performance.

Although our results on DM and ADNI datasets demonstrate the great potential for integrating MRI and EHR data to improve the risk prediction performance of brain degeneration; however, there are some limitations of the proposed method.

Considering the inherent bias of DM and ADNI datasets (Pipitone et al., 2014), it is essential to investigate the performance of multimodal learning models on diversified data, such as more than two modalities of data, to generalize the prediction ability of our method in clinical applications. The number and diversity of datasets are still critical bottlenecks for the performance improvement of the proposed learning model. With a large number of diversified datasets, the prediction performance gain can be obtained by diversified feature enhancements. In addition, the internally collected DM datasets with different patient groups are not well balanced, which may impact the evaluation of the sensitivity gap. With limited DM datasets, the proposed method has improved the prediction of brain degeneration by classifying the three levels of DM patients. Therefore, more extensive studies will be necessary to validate the generalization ability of the proposed attention-based learning model despite our promising preliminary results from internal DM and public ADNI datasets.

In this study, we only select limited features (e.g., 17 features of DM patients and 11 features of ADNI patients) to create the EHR data, the extensive study to rely on MRI image features to guide the selection of more EHR features needs a deep exploration.

With the advent of deep transfer learning technology (Grassi et al., 2019; Bae et al., 2021; Alanazi et al., 2022), the performance of the proposed framework may be optimized by using other modalities of data, such as functional MRI and molecular imaging by mass spectrometry to provide more efficient and accurate predictions. Our method can aid the early diagnosis of brain degeneration and improve the diagnosis workflow. Meanwhile, our proposed method has great potential to be translated to predict the risk of other diseases. Based on other modalities of data, it incorporates more data properties to construct multimodal learning strategies for the prediction of other diseases, such as melanoma and multiple sclerosis (Huang et al., 2020).

## 5. Conclusion

In this study, we propose a novel attention–based learning framework by incorporating MRI images and EHR data, to improve the precision of brain degeneration diagnosis. Compared to the single-modal features, the optimal global feature representations extracted from MRI features and EHR features play an essential role in the final decisions of the learning model. Through the study, the proposed method is superior to the MRI-only, EHR-only, and typical multimodal fusion methods for predicting brain degeneration.

We deployed suitable attention mechanisms for each module of our framework to extract related information to improve the performance model, which may also be applied to other prediction tasks. Meanwhile, we should focus on the multi-head attention mechanism with different head numbers, which is usually valuable and practical to enhance the final elaborating representations from multimodal data. The designed encoder and decoder modules only depend on self-attention mechanisms, which are flexible to further applications and extensions.

In general, the proposed method provides an efficient aid for clinical diagnosis and early prevention of brain degeneration by extracting disease-oriented related information based on medical images and non-image clinical context information.

## Data availability statement

The original contributions presented in this study are included in the article/supplementary material, further inquiries can be directed to the corresponding author.

## Ethics statement

The studies involving human participants were reviewed and approved by Affiliated Zhongshan Hospital of Dalian University, Department of Radiology. The patients/participants provided their written informed consent to participate in this study.

## Author contributions

XS and WG contributed to the conception and design of the study. XS performed the data analysis and wrote the first draft of the manuscript. All authors contributed to the manuscript revision, read, and approved the submitted version.
