# Corrigendum: Toward attention-based learning to predict the risk of brain degeneration with multimodal medical data

**DOI:** 10.3389/fnins.2023.1153816

**Published:** 2023-03-07

**Authors:** Xiaofei Sun, Weiwei Guo, Jing Shen

**Affiliations:** ^1^Department of Electrical and Electronic Engineering, The University of Hong Kong, Hong Kong, Hong Kong SAR, China; ^2^EchoX Technology Limited, Hong Kong, Hong Kong SAR, China; ^3^Department of Radiology, Affiliated Zhongshan Hospital of Dalian University, Dalian, Liaoning, China

**Keywords:** risk prediction of brain degeneration, multimodal medical data, multimodal learning, self-attention mechanism, cross-attention mechanism

In the published article, there was an error in affiliations (3, 4). Instead of “[^3^Graduate School, Tianjin Medical University, Tianjin, China, ^4^Department of Radiology, Affiliated Zhongshan Hospital of Dalian University, Dalian, Liaoning, China],” it should be “[^3^Department of Radiology, Affiliated Zhongshan Hospital of Dalian University, Dalian, Liaoning, China].”

The authors apologize for this error and state that this does not change the scientific conclusions of the article in any way. The original article has been updated.

